# An IoT-Based Solution for Monitoring a Fleet of Educational Buildings Focusing on Energy Efficiency

**DOI:** 10.3390/s17102296

**Published:** 2017-10-10

**Authors:** Dimitrios Amaxilatis, Orestis Akrivopoulos, Georgios Mylonas, Ioannis Chatzigiannakis

**Affiliations:** 1 Computer Technology Institute and Press “Diophantus”, Patras 26504, Greece; mylonasg@cti.gr; 2 Sparkworks Ltd., Altrincham WA14 1RP, UK; akribopo@sparkworks.net; 3 Department of Computer, Control and Informatics Engineering, La Sapienza University, Rome 00185, Italy; ichatz@dis.uniroma1.it

**Keywords:** Internet of Things, energy efficiency, educational buildings, non-residential buildings, real-time monitoring, education, behavioral change, open-source, cloud computing, evaluation

## Abstract

Raising awareness among young people and changing their behaviour and habits concerning energy usage is key to achieving sustained energy saving. Additionally, young people are very sensitive to environmental protection so raising awareness among children is much easier than with any other group of citizens. This work examines ways to create an innovative Information & Communication Technologies (ICT) ecosystem (including web-based, mobile, social and sensing elements) tailored specifically for school environments, taking into account both the users (faculty, staff, students, parents) and school buildings, thus motivating and supporting young citizens’ behavioural change to achieve greater energy efficiency. A mixture of open-source IoT hardware and proprietary platforms on the infrastructure level, are currently being utilized for monitoring a fleet of 18 educational buildings across 3 countries, comprising over 700 IoT monitoring points. Hereon presented is the system’s high-level architecture, as well as several aspects of its implementation, related to the application domain of educational building monitoring and energy efficiency. The system is developed based on open-source technologies and services in order to make it capable of providing open IT-infrastructure and support from different commercial hardware/sensor vendors as well as open-source solutions. The system presented can be used to develop and offer new app-based solutions that can be used either for educational purposes or for managing the energy efficiency of the building. The system is replicable and adaptable to settings that may be different than the scenarios envisioned here (e.g., targeting different climate zones), different IT infrastructures and can be easily extended to accommodate integration with other systems. The overall performance of the system is evaluated in real-world environment in terms of scalability, responsiveness and simplicity.

## 1. Introduction

Efforts to decrease energy consumption as a society have placed strong focus on energy-related issues inside buildings, as well as in other sectors such as transportation and industry. A very interesting subset of buildings, in this respect, is the nonresidential buildings sector; the buildings in which, among other activities, we work and study, comprise a large part of our overall energy consumption. However, although a large number of studies have focused on workplaces, office buildings, and retail spaces, the educational buildings sector has not attracted equivalent attention by the research community, at least in relation to its importance.

In the EU, educational buildings constitute 17% of the non-residential building stock (in $m^{2}$) [1], the third largest sector after wholesale/retail (28%), and offices (23%). Moreover, evidence shows that a focus on energy consumption in schools yields multiple benefits, along with educational excellence and a healthy learning environment [2]. It is thus the ideal candidate for applying IoT technologies in order to provide a foundation for energy savings-related activities through monitoring. Moreover, this sector has several unique and interesting features; firstly, educational organizations (e.g., ministries) manage a large number of buildings. Secondly, in many cases in existing installations there is little need to have a complex Heating, ventilation, and air conditioning (HVAC) infrastructure, e.g., in several European countries, school buildings are often quite old, have central heating or cooling, having limited control possibilities, etc. Thirdly, such entities wish to avoid vendor lock-in, while a single solution in terms of infrastructure is impossible since each educational building has different characteristics. Moreover, another interesting point in this case, is that data from such systems could be utilized in a number of ways in an educational context.

Another interesting aspect regarding the energy efficiency of schools is the fact that historically, energy expenses in schools have been treated as relatively fixed and inevitable. Evidence shows that a focus on energy use in schools yields an array of important rewards in concert with educational excellence and a healthful learning environment [2]. Since energy costs are the second largest expenditure within school district budgets, exceeded only by personnel costs [3], significant savings can be carved out, if energy consumption can be reduced. We expect that an IoT infrastructure can help improve the efficacy of a behaviour-based approach by actively involving the school staff (e.g., school directors, building managers, custodial staff and teachers) in fostering a culture of energy conservation. If empowered to do so, building managers and custodial staff can offer critical insights about ways to lower a building’s energy footprint through effectively managing building operations [4,5,6]. It is important to point out that the EU considers environmental education one of the most prominent instruments to influence human behavior towards environmentally sustainable patterns.

Having the above in mind, in this paper a system is provided that covers the aforementioned needs, serving as a platform for monitoring a fleet of educational buildings, focused on energy monitoring and enabling a number of different application and implementation directions, based on an IoT infrastructure deployed inside such buildings. In short, a system is needed with the following properties:

- *openness*, to support a number of different IoT ecosystems,
- *versatility*, to support different application domains, e.g., energy efficiency and educational scenarios,
- *scalability*, to support a very large number of buildings and IoT sensing endpoints,
- *up-to-date support of modern practices* in the design of the system, i.e., cloud-based solutions, easy deployment, etc.

In this work, a system is presented with the above properties, which aims to offer a complete solution in this respect. It is currently being used in 18 educational buildings in multiple European countries, in the context of the GAIA research project [7,8]. The project’s goal is to promote sustainability awareness and energy efficiency in an educational setting, utilizing real-world data inside class lectures, educational labs, building monitoring and gamification. For this goal, it utilizes a number of different IoT technologies as infrastructure installed inside these school buildings, while it follows an open, cloud-based approach for its implementation, which enables the development of applications on top of it. Remark that the IoT devices that were installed are either open-source hardware of very low cost (less than 50$ per device) or are off-the-shelf products that are also low cost.

Since the community is gradually moving from vertical single purpose solutions to multipurpose and collaborative applications interacting across industry verticals, organizations and people, a cloud-based approach is utilized. The necessity for data collection, storage and availability across large areas, the demand for uninterrupted services even with intermittent cloud connectivity and resource constrained devices, along with the necessity of sometimes near-real-time data processing in an optimal manner, create a set of challenges where only holistic solutions may apply.

The system presented here provides a uniform environment where edge and cloud resources can be interchanged without the need for additional development for managing the infrastructure. Domain experts focus on specifying the rules for data-driven processing and event-based response and let our platform automatically tune the infrastructure for high-performance and high-availability. The resulting system continues to operate even when certain parts of the IoT domain become disconnected or experience periods of low-bandwidth. We provide a performance evaluation, having also discussed several aspects of the targeted application domain. Such application requirements have shaped our selection of evaluation criteria: we showcase that our system is efficient and highly responsive, while also being able to enable the development of a set of applications that augment its functionality.

Special emphasis has been given towards developing a long-term cooperation between researchers, users and other relevant stakeholders to bring users and technology into an open design process in real life environments, supporting long-term cooperation, co-creative research and a continuous, long-hauled study of user experiences. As part of this approach, the system is developed based on open-source technologies and services. Specific decisions are made in terms of the overall system architecture in order to make the system open to provide an open IT-infrastructure capable of supporting different commercial hardware/sensor vendors as well as open-source solutions. The system can be used to develop and offer new app-based solutions in all fields of the Future Internet related to the energy sector. As a result, the system is replicable and adaptable to settings that may be different than the scenarios envisioned here (e.g., targeting different climate zones) and different IT infrastructures.

The rest of the work is organized as follows: in Section 2, relevant previous work is discussed, in Section 3 an overview of the system is provided, and it goes through a discussion of specifics of its implementation. Section 4 presents an evaluation of the system in terms of performance, while Section 5 discusses aspects of integration and application development over our platform. In Section 6 the paper concludes along with a discussion on future directions.

## 2. Related Work

Energy efficiency and savings in buildings are one of the popular issues in the smart cities, IoT, and ICT overall research agenda. In this respect, the European Union has been especially active in promoting energy efficiency in buildings. Build Up [9] is an EU-specific portal for gathering all types of resources for energy efficiency in buildings, e.g., project results, national regulations, etc., while also funding a multitude of research projects on the subject.

A large body of recent work addresses issues such as the installation and management of the IoT infrastructure inside buildings. The work of [10] discusses the strategic selection and placement of sensors inside buildings in order to provide a good solution in terms of observability with respect to building energy management. Brick [11] discusses a uniform schema for representing metadata in buildings. In [12] a framework for automatically classifying, naming and managing sensors based on active learning from sensor metadata is presented. [13] studies the design of a large-scale IoT system for smart grid application, which constitutes of a large number of home users and has the requirement of fast response time. [14] performs a literature survey on smart grid testbeds around the world, and presents the main accomplishments toward realizing a smart grid testbed able to monitor, analyze, and evaluate smart grid communication network design and control mechanisms, and test the suitability of various communications networks for both residential and commercial buildings. [15] describes a testbed in a residential building which was setup to evaluate the degree up to which, energy can be throttled for energy management purposes without affecting the end user’s comfort level.

The IoT infrastructure design and installation presented in this paper was largely dictated by the chosen application domain (education), and by the fact that although each building has unique features, the structure in classrooms, similar operational characteristics and the requirement to have a similar (in size and composition) installation in all buildings to serve educational purposes led to a somewhat homogeneous IoT infrastructure. There is also a growing list of works offering open datasets from all types of buildings, e.g., [16] and [17]. This a direction that is important to look into in the near future, since the GAIA platform has already, in some cases, years’ worth of school building data.

Another interesting future perspective are the emerging trends of Fog and edge computing that provides computational, storage, and control resources in an intermediate layer between end-user devices and cloud computing datacenters. The physical proximity of fog infrastructure with the resource-bound last-mile sensors of any IoT-related application, allows limited latency, less bandwidth consumption, as well as elevated degrees of reliability and security. This approach extends the cloud computing paradigm by migrating data processing closer to the production site, accelerates system responsiveness to events along with its overall awareness, by eliminating the data round-trip to the cloud. In [18] the benefits of this approach is demonstrated for new applications in the realms of the IoE, Smart City, Industry 4.0, and Big Data Streaming, while introducing new open issues. A system for monitoring the performance of buildings that follows the Fog Computing paradigm where the sensor resources, as well as the intermediate layers between embedded devices and cloud computing datacenters, participate by providing computational, storage, and control is provided in [19].

In [20], a system that utilizes similar components is discussed, and although it does not focus solely on educational buildings, it provides insights derived from initial results with respect to a deployment inside a university building. Regarding other aspects related to the work presented here, [21] the open-source hardware design of the IoT nodes utilized in the majority of the buildings that were used to evaluate the performance of the system presented in this work, are discussed. In addition, [22] the design and implementation of an energy savings recommendation component that utilizes the IoT data of the said buildings is also discussed. Although the work has focused on educational buildings, a number of existing works on energy savings in residential/commercial buildings could be also implemented. As an example, [23] discusses the effect of consumption feedback produced from disaggregated home device data; the system presented here could be utilized to implement such an approach. As another example, [24] discusses an approach for energy savings related to occupancy in commercial buildings. In this work, the evaluation is based on occupancy data produced by activity sensors inside classrooms. A demand response management (DRM) scheme is proposed in [25] and a residential smart grid testbed is constructed to evaluate the peak load reduction and verify the effectiveness and efficiency of different DRM schemes.

Moreover, several approaches have been proposed in order to address the potentially huge number of sensor data arriving from the IoT domain, each one of them applied in different parts of the network architecture [26,27]. Starting from low-end devices, the approach of in-network aggregation and data management has been proposed where sensor devices follow local coordination schemes in order to combine data coming from different sources and/or within the same time period based on similarities identified using data analysis [28,29,30]. [31] discusses the design of Internet of Things (IoT) for a flexible Building energy management (BEM) with the backend analytic that performs analysis on energy usage pattern and the wastage. For an overview of different building automation technologies, see [32]. For an overview of different techniques and existing protocols, see [33]. While such mechanisms are not performed by the IoT devices, the system discussed here can be combined with such an approach.

## 3. Educational Building Specific Implementation

In order to evaluate the systems’s performance in a real-world environment, we look into the broad domain of people-centric applications [34] that will facilitate the educational sector towards improving the energy efficiency of school buildings. We envision an IoT ecosystem that is composed of a variety of business players that collaborate towards bringing together a diverse set of devices for real-time monitoring and management of school buildings. To do so, we work by following the concept and scope of the IoT as defined by the International Telecommunication Union Standardization Sector (ITU-T) in 2012 [35].

The educational sector presents a very interesting and important case for the monitoring and management of institutional buildings, by having a very large number of buildings to operate, situated in a very fragmented manner. In national educational systems we have literally thousands of buildings spread throughout a country, usually, with very different characteristics in terms of construction, age, size, etc. It is reasonable to expect a diverse set of device providers working under the same interoperability framework. Due to this, we are hardware independent: sensors from different manufacturers inter-operate with our system.

**Figure 1:**
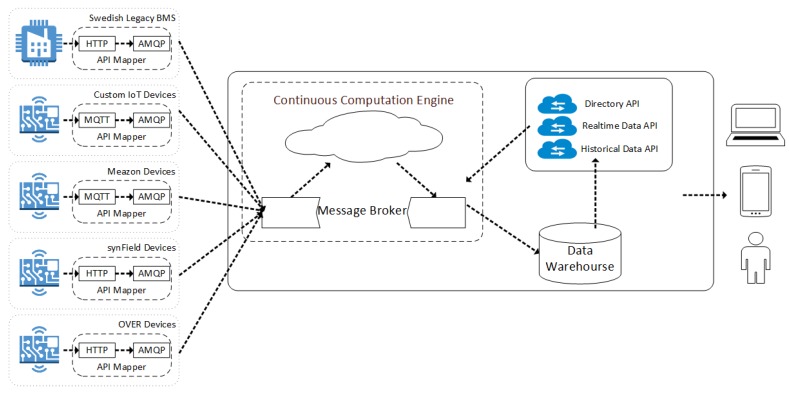
Educational building-specific IoT architecture.

Our platform enables direct comparisons of energy efficiency between buildings and cities, carefully taking into account many environmental parameters. We integrate the diverse capabilities of IoT devices and provide open interfaces to application developers. Our platform supports different end-user groups that inherently exist in the educational sector: students, educators, building administrators and other administrative staff. Our platform’s interfaces provide access to the available information in a way that suits all of these end-users groups. In some cases unifying different school buildings into a single view is necessary to make interaction simpler and data visualization more natural and create an environment that conveys valuable insights and clear actions related to general or specific aspects of the participating building ecosystem. For a graphical representation of the components used in the specific implementation fo the educational buildings see Figure 1.

Regarding the actual deployment of our prototype, thus far 18 school buildings have been involved (see Table 1), spread in 3 countries (Greece, Italy, Sweden), covering a range of local climatic conditions and educational levels (primary, secondary, high school and university). Currently, electricity consumption meters are installed in all of these buildings, along with sensors monitoring indoor and outdoor conditions as described above. The vast majority of the rooms monitored are used for teaching purposes and the rest for other activities like teacher/staff rooms, etc. The year of construction of these buildings ranges from 1950 to 2000.

### 3.1. End-Device Layer

In each of the 18 buildings participating in our pilot study, we deployed sensor devices that measure (a) the overall power consumption of the building, (b) the environmental comfort within each individual class (see below for more details), and (c) the weather conditions and air pollution levels in each building. These devices can be split into three different categories based on their origin and operation type. In general, we use (i) custom made IoT devices that communicate using an IEEE 802.15.4 local network, (ii) proprietary off-the-self IoT devices that communicate using IEEE 802.11 and 3G in areas that we cannot easily connect to, and (iii) sensors from legacy Building Management Systems (BMS) that are already installed in a number of school buildings. Most indoor IoT nodes form IEEE 802.15.4 networks (Zigbee or plain) and communicate with their respective edge devices by establishing ad hoc multihop bidirectional trees, set up at the time of the deployment and maintained throughout the network lifetime. The outdoor nodes are connected via Power Over Ethernet cables to transfer both electricity and maintain communication over a single cable, while in some other cases we also used IEEE 802.11 and supplied the weather stations with batteries and solar panels to harvest energy from the sun. On the transport and session layers, the devices communicate using either a custom protocol or Zigbee for the discovery of resources and transmission of measurements. In the rest of this section, we provide some more details on the categories of devices we have integrated.

#### 3.1.1. Custom IoT Devices

**Power Consumption** The *power consumption meters* installed measure the apparent power and average power consumption of a school building. Meters are situated on the general electricity distribution board of each such building to measure each one of the three-phase power supply of the building. These devices are equipped with XBee modules in order to access the IEEE 802.15.4 network and transmit the measurements to cloud services via the custom made gateway nodes. For more details regarding the design and technical specification of the devices see [21].

**Environmental Comfort** The *environmental comfort meters* measure various aspects affecting the well-being of the building’s inhabitants, such as thermal (satisfaction with surrounding thermal conditions), visual (perception of available light) comfort and overall noise exposure. We also monitor room occupancy using passive infrared sensors (PIR). These devices are also equipped with XBee modules in order to access the IEEE 802.15.4 network and transmit the measurements to the cloud services via our custom-made gateways. For more details regarding the design and technical specification of the devices see [21].

**Weather and Atmosphere Stations** These provide information on the outdoor atmospheric conditions including precipitation levels, wind speed and direction. The *atmospheric meters* monitor atmospheric pressure and the concentration of selected pollutants, to provide insights on the pollution levels near school buildings. These devices communicate with our system directly via Ethernet or WiFi and are powered using Power-Over-Ethernet or are plugged into the sockets of the building when available. For more details regarding the design and technical specification of the devices see [21].

#### 3.1.2. Proprietary Devices

**Meazon** In certain locations where the installation of our custom devices was not feasible due to connectivity or other restrictions, we have installed a number of Meazon power meters and sensors. These sensors communicate with Meazon’s proprietary data infrastructure and their data are then pushed to our platform. On the hardware side, these devices communicate using Zigbee to a central gateway device that is either connected to the Internet via Ethernet or use 3G in order to communicate directly with Meazon’s proprietary cloud services.

**synField** Similarly, in some of the buildings instead of our custom weather stations, we used some off-the-self synField weather stations that offered us WiFi connectivity to avoid installing additional cables on the roofs of the buildings, as well as energy harvesting via solar panels. In this case, the weather stations communicate via WiFi directly to proprietary cloud services.

#### 3.1.3. Legacy Installations

**BMS** In one of the schools involved (a large technical high school/college) a BMS was already in place, utilized by the building manager and other technical staff to monitor and control several aspects of the day-to-day business. However, this system provided little to none standard interfaces to external systems. To integrate its infrastructure to our system a custom application was developed to poll periodically the collected data directly from the application’s database and transmit the data to our platform.

### 3.2. Data Access and Acquisition

Our platform provides a unified API for retrieving data from multiple sites and multiple hardware platforms with transparency. Each hardware device integrated in the platform is mapped to a *resource*. Resources are self-described *Entities* and are also software/hardware agnostic. The Data API acts as a wrapper function and hides much of the lower-level plumbing of hardware specific API calls for querying and retrieving data, providing a common API for retrieving historical or real-time data from resources.

To facilitate integration between the existing hardware and software technologies, the exchange of the information occurs through *API Mappers*. The API Mapper acts as a translation proxy for data acquisition and it is responsible for polling the devices infrastructure through proprietary APIs and translating the received measurements in a ready to process form for the platform. In general, the API Mapper transforms data to and from the API. The data input type can be, based on each device’s capabilities, either poll based and/or push-based. In more detail, the API Mapper is capable of receiving data from the IoT devices but also of sending messages/commands to the devices. Furthermore, according to the system design, the API Mappers introduce scalability and modularity in the platform. Our solution offers two separate types of API Mappers for integration with external services and to retrieve IoT sensor data: (a) Polling API Mapper and (b) Message Queue API Mapper. Both solutions will be used in order to integrate with data originating from IoT installation.

The first solution (Polling API Mapper) is based on polling. A usage example is the following: weather stations are installed in a subset of school buildings of the project. Data produced by such stations are accessible through the weather application that provides historical information through a RESTful API provided by the weather backend. In order to integrate them into the system presented here, the weather API Mapper was implemented for the weather API based on the Polling API Mapper. The weather API is polled every fixed number of minutes (5) for updated data. When new data is found, it is formatted to the internal format of the platform and forwarded to the Processing/Analytics engine for processing and analysis using the AMQP protocol. The data is then processed and can be accessed by the system’s Data API. Similar implementations based on the Polling API Mapper will be used to integrate IoT devices provided by third parties and the existing BMSs installed in school buildings.

The second solution is used when a pub/sub solution exists in the external service that is going to be integrated. In that case, the external service is capable of publishing the IoT data (generated or gathered) to an MQTT endpoint. The API Mapper is then able to receive new measurements asynchronously and format them to the internal format of our platform. The data is then forwarded to the system’s Processing/Analytics engine for processing and analysis using the AMQP protocol. The data is then processed and can be accessed from the system’s Data API. Messages inside the MQTT broker can be transferred in multiple formats ranging from plain text to any open or proprietary protocol. In our case messages are transmitted in plain text following a simple format: the topic of the message refers to the device and sensor that generated the message while the actual payload represents the value generated. For example, if a sensor with a hardware (MAC) address 124B00061ED466 publishes a temperature value of 20 degrees, the topic is 124B00061ED466/temperature and the message 20. All sensors forward their measurements periodically (every 30 s) or on events (i.e., when motion is detected) and the API mapper receives and forwards them to the processing engine.

## 4. Evaluation

The evaluation of the system’s operation focuses on the following areas: (1) data processing, (2) data access, and, (3) data analysis and statistics. These areas can adversely affect the performance and perception of a system since users need rich data, easily and quickly accessible and real-time information to better understand how their actions affect the building usage. This is more crucial when the data is used in the educational context, i.e., during courses.

### 4.1. Data Analysis

**Figure 2:**
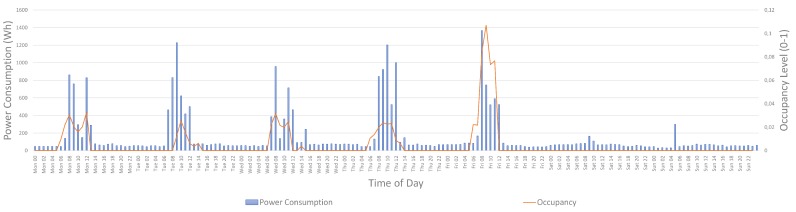
Four-week (May 2017) average occupancy levels in four different school buildings.

To better present the operation and capabilities of the system, an analysis of the average weekly occupancy of 4 distinct school buildings during May 2017 is presented in this section. Remark that this analysis is similar to the work presented in [36]. Figure 2 depicts the occupancy levels of the whole building, as an aggregated occupancy of all the rooms in which a smart motion sensors is installed. All four buildings are elementary schools that follow the same academic schedule with activities starting from 08:00 until 13:30. Remark that the building of “School 1” is also used by a technical school that is used in the afternoon. The data presented are ranged from 0 (no motion detected during this time interval) to 1 (constant motion was detected during the whole time period). This graph shows the actual active hours of the schools that are commonly ranged between 7:00 and 15:00 during weekdays. It also depicts the clear differences between schools of different levels, e.g., the case of “School 1” where a Technical School has class hours also in the afternoon.

**Figure 3:**
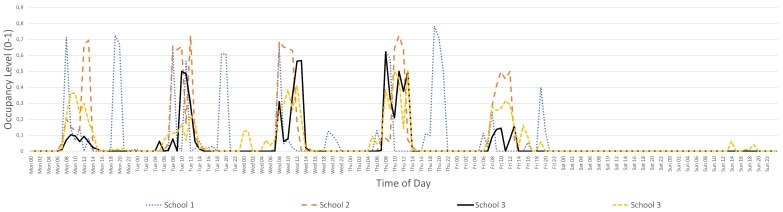
Four-week (May 2017) average power consumption and occupancy levels in a specific school building.

Similarly, Figure 3 presents the average power consumption of a school as it is measured by our system, versus the occupancy of the building. From the graph, it is clear that the school building consumes power mainly when it is occupied during the weekdays.

### 4.2. Data Access

**Figure 4:**
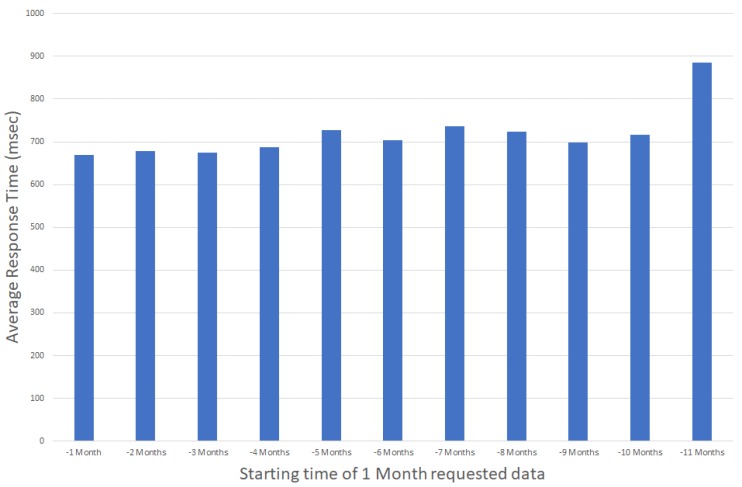
Average Response Time for accessing one month data for the past year (daily aggregated values).

**Figure 5:**
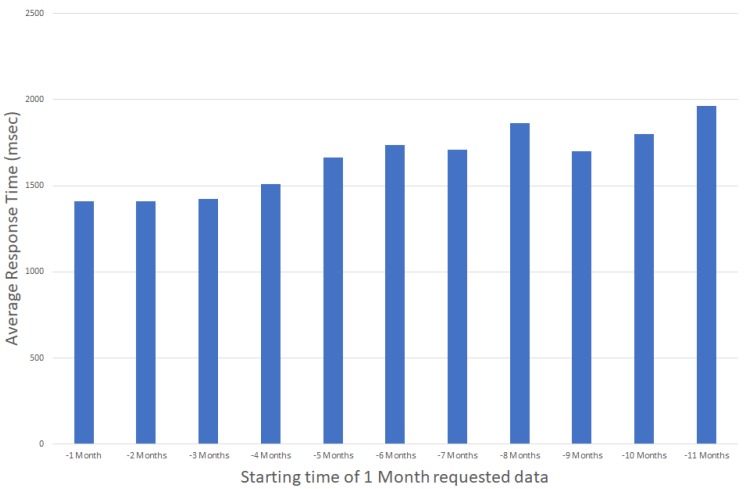
Average Response Time for accessing one month data for the past year (hourly aggregated values).

Accessing historical data is crucial for building monitoring applications, e.g., when comparing historical data from different time spans and building areas. In such use cases, it is important that an IoT service is capable of providing these data without delays, independently of the targeted time interval. As discussed in [37], application response times larger than 10 s tend to make users lose their attention in the given task, while a 1 s response time is considered the limit for users that are freely navigating an application without waiting for the application’s response. In that context, when presenting power consumption statistics, e.g., over the past year, it is important to be able to retrieve and present the stored values within one second, independently of the requested interval (latest values versus older values). Figure 4 and Figure 5 present the average retrieval times for accessing historical data of a one month duration for the past 12 months, observing minimal differences in the access times independent of the period requested.

**Figure 6:**
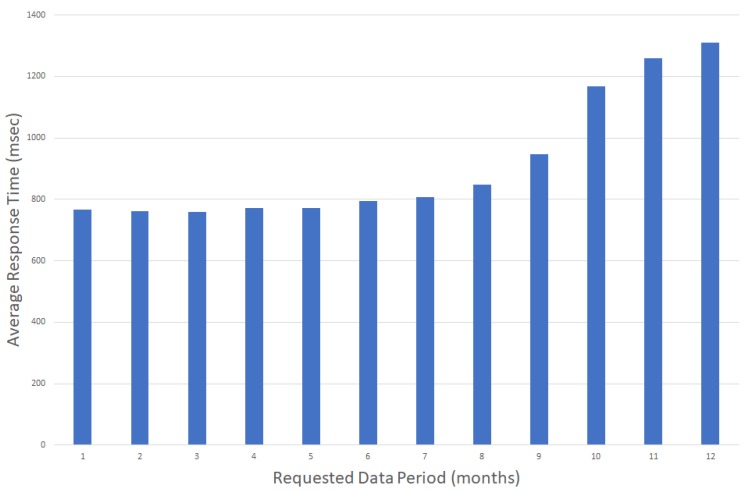
Average Response Time for variable time periods ranging from one to 12 months.

Note that, based on the data available from the graphs, the system’s response time is independent of the actual time interval while it is actually dependent on the amount of data requested. This is more clear in Figure 6, where response times tend to increase as the response times increase when time periods of more than 9 months of data are requested.

### 4.3. Data Processing

Another important characteristic for evaluating the system’s performance is the load of data that the system is able to process at any given time. With the current setup, the fleet of buildings in the system produces an average of 25 measurements per second. The data processing topology currently runs on a single core virtual machine (on an Intel®Core™i5-3340 CPU running at 3.10GHz) with 4GB of RAM. With this configuration and setup, the system is capable of processing up to 500 measurements per second. To increase the number of measurements, the system can support two different options:

- Increase the computing power of the virtual machine, by assigning it to a more powerful host or giving it access to more resources from the host machine.
- Deploy a second instance of the processing topology that is capable of consuming the same number of measurements to reach the required data processing rates.

Based on the nature of sensors deployed, the input data require three different types of aggregation: (1) *averaging* for sensors such as temperature or relative humidity, (2) *total* for sensors such as rain height levels, and (3) *power consumption estimation* based on the electrical current values received from the installation. Each type of processing requires a different type of aggregation processing and as a result has a different average execution latency, presented in Table 2.

## 5. Integration/App Development

As previously mentioned, the system offers a rich set of APIs to access the information of the deployed sensors as well as the data collected in real time. These APIs have been used to develop multiple applications that are designed to help the building managers, teachers and students in their day-to-day activities in the school buildings (educational or not). Among others, third parties have developed a building management application (BMS) [38], a participatory sensing application, as well as a set of in class activities accompanied by sensing and visualization tools that help students better understand their environment and the natural effects monitored.

### 5.1. BMS

In order to monitor the energy efficiency of the Educational Buildings, a multi-school BMS System has been developed using the API exposed by the system developed here. Using the BMS System the building manager will be able to inspect real-time energy usage, inspect results from a comparison with similar buildings or with the same building in other time spans (e.g. previous years), along with comments and receive energy efficiency recommendations. It is also important to mention that building managers are able to communicate using the BMS System in order to enhance their knowledge of actions that can lead to energy savings.

### 5.2. Participatory Sensing

As a way to complement existing IoT infrastructure and provide additional data from the school buildings monitored by the system, an Android application was developed by a third party to provide participatory sensing capabilities. Smartphones and tablets are used to provide additional readings from inside the school building, e.g., luminosity or noise levels, while participants can also manually enter data such as electricity meter readings. The teacher can initiate participatory sensing sessions during the courses from the main portal of the project and then students can use phones and tablets to gather data in real time and then review them in class.

### 5.3. In-class Activities and Gamification

In order to augment the application set developed for the system and extend its educational focus, several applications have been implemented to support educational content based on the data produced by the IoT infrastructure inside the monitored school buildings. These are based on the fact that students are more driven to engage in class activities regarding sustainability and energy efficiency when the data utilized originates from their environment and are near real-time. The activities are built around a DIY sensor kit, which the students use to build a small interactive installation that visualizes environmental and energy consumption data from school classrooms. In more detail, students are able to start from building a simple electrical circuit, using resistors, LEDs and motors, and move progressively to running and coding simple application scripts in Python that communicate with our system to retrieve the sensed values of their surroundings. These values are visualized using the LEDs or the motors and students can then see how their actions inside the classrooms and their school affects their surroundings.

## 6. Conclusions

The total amount of energy spent in our everyday life (residential use, personal transportation, etc.) is quite significant, reaching up to 40% of the world’s total energy usage. In this context, reducing energy use in buildings by introducing new technologies is very challenging; across Europe, the rates of new buildings construction as well as the rates of renovation of existing buildings are both generally very low. Similarly, energy consumption in transportation is growing annually, also making it very challenging to save energy in this sector. Therefore, it is evident that, in order to achieve the ambitious energy and climate targets for 2020 and beyond, we need a change in citizens’ behaviour and consumption practices. Reports indicate that citizens making efficient use of energy in their everyday life can lead to large energy and financial savings and potentially to a substantially positive environmental impact.

This work adopts a holistic approach towards designing an efficient system together with a sustainable vision, utilising IoT infrastructures combined with a diverse set of feedback and interface mechanisms, in order to engage its end-users in long-term behavioural changes. The system is deployed in school buildings in several countries representing south, central and northern European areas, with both cultural and geographical diversity. The system is developed based on open-source technologies and services in order to make the system open to provide an open IT-infrastructure capable of supporting different commercial hardware/sensor vendors as well as open-source solutions. The system presented can be used to develop and offer new app-based solutions that can be used either for educational purposes or for managing the energy efficiency of the building. The system is replicable and adaptable to settings that may be different than the scenarios envisioned here (e.g., targeting different climate zones), different IT infrastructures and can be easily extended to accommodate integration with other systems.

The performance evaluation presented is evaluated in real-world environment comprised of 13 school buildings and including 700 IoT endpoints. The overall performance analysis demonstrate its scalability, responsiveness and versatility in accomodating different application needs. The results show that the system is highly efficient, by handling the respective building data loads almost in real time using typical server resources, and responsive, with an application response time less than 2 s, despite the large scale of the IoT deployment inside the educational buildings monitored. In particular the system is capable of processing the data arriving from all 13 buildings using a single core virtual machine (on an Intel®Core™i5-3340 CPU running at 3.10GHz) with 4GB of RAM. With this configuration and setup, the system is capable of processing up to 500 measurements per second. Interestingly, the use of open-source technologies to create an open design platform does not limit the overall performance of the system. The system’s usage in order to accomodate a larger number of buildings can rely on additional resources at the cloud level where the virtual machine is executed. Alternatively, by adopting the Fog computing paradigm, see e.g. [19], separate virtual machines can be distributed to each building that act as the IoT gateways and accomodate the needs of each separate building without increasing the overall costs of the cloud infrastructure.

A key requirement of a system targeting the educational sector is the ability to address the needs of diverse user groups such as building managers, teachers and students in their day-to-day activities in the school buildings (educational or not). The system presented here offers a rich set of APIs to access the information of the deployed sensors as well as the data collected in real time. A number of different applications were presented that target building managers (e.g., such as the BMS application), applications that augment the in-class activities and extend the educational content based on the data produced by the IoT infrastructure as well as mobile applications that offer participatory sensing capabilities that allow the teachers to engage students in diverse activities that span also outside school hours. It is therefore evident that providing a concrete group of APIs is critical for supporing the development of multiple applications that are designed to help the different types of users and also allow third-party applications.

In terms of future work, it is important to further extend the capabilities of the system, mostly through extending the set of applications built around it, e.g., gamification and other applications supporting educational aspects. Another direction of work is to provide open datasets, produced by the system’s application in real-life settings in the educational domain. These datasets will allow the buildings’ operations analysis not only in terms of energy consumption but also in terms of ambient environmental conditions with possible connections to the educational activities. Remark that such data-driven analysis of the operation of the educational sector does not exist at this point. Providing evidence of such aspects of the educational activities is crucial to improving the overall quality and performance of schools. Finally, extending the IoT infrastructure in order to provide data through the integration of additional school buildings is also an important future work direction.

## Figures and Tables

**Table 1:** Key facts of our deployment.

Parameter	#	Description
Educational Buildings	18	13 Greece
		4 Italy
		1 Sweden
Sensing Points	855	≥ five sensors per device
Students	5500	students in all levels
Teachers	900	teachers in all levels
Sensing Rate	0 s	classroom sensors

**Table 2:** Execution Latency statistics for the three different aggregation types used in our system.

Aggregation Type	Execute Latency (ms)	Measurements (%)
Average	0.608	86.4
Total	0.799	0.9
Power Consumption	0.329	12.7

## References

[1] Economidou M., Atanasiu B., Despret C., Maio J., Nolte I., Rapf O., Laustsen J., Ruyssevelt P., Staniaszek D., Strong D. (2011). Europe’s Buildings under the Microscope: A Country-by-Country Review of the Energy Performance of Buildings.

[2] Crosby K., Metzger A.B. (2012). Powering Down: A toolkit for Behavior-Based Energy Conservation in K-12 Schools.

[3] (2008). Energy Star Building Upgrade Manual.

[4] Cross J.E. (2012). Shifting Organizational Culture—Innovation for Energy Conservation.

[5] Schelly C., Cross J.E., Franzen W.S., Hall P., Reeve S. (2011). Reducing Energy Consumption and Creating a Conservation Culture in Organizations: A Case Study of One Public School District. Environ. Behav..

[6] Schelly C., Cross J.E., Franzen W.S., Hall P., Reeve S. (2012). How to Go Green: Creating a Conservation Culture in a Public High School through Education, Modeling, and Communication. J. Environ. Educ..

[7] Mylonas G., Amaxilatis D., Helen L., Zahariadis T., Zacharioudakis E., Hofstaetter J., Friedl A., Paganelli F., Cuffaro G., Lerch J. Addressing Behavioral Change towards Energy Efficiency in European Educational Buildings. Proceedings of the Global Internet of Things Summit (GIoTS).

[8] GAIA Project. http://gaia-project.eu.

[9] European Web Portal for Energy Efficiency in Buildings. http://www.buildup.eu.

[10] Agarwal A., Munigala V., Ramamritham K. (2016). Observability: A Principled Approach to Provisioning Sensors in Buildings. Proceedings of the 3rd ACM International Conference on Systems for Energy-Efficient Built Environments.

[11] Balaji B., Bhattacharya A., Fierro G., Gao J., Gluck J., Hong D., Johansen A., Koh J., Ploennigs J., Agarwal Y. (2016). Brick: Towards a Unified Metadata Schema For Buildings. Proceedings of the 3rd ACM International Conference on Systems for Energy-Efficient Built Environments.

[12] Balaji B., Verma C., Narayanaswamy B., Agarwal Y. (2015). Zodiac: Organizing Large Deployment of Sensors to Create Reusable Applications for Buildings. Proceedings of the 2nd ACM International Conference on Embedded Systems for Energy-Efficient Built Environments.

[13] Viswanath S.K., Yuen C., Tushar W., Li W.T., Wen C.K., Hu K., Chen C., Liu X. (2016). System Design of the Internet of Things for Residential Smart Grid. Wirel. Commun..

[14] Tushar W., Yuen C., Chai B., Huang S., Wood K.L., Kerk S.G., Yang Z. (2016). Smart Grid Testbed for Demand Focused Energy Management in End User Environments. IEEE Wirel. Commun..

[15] Li W.T., Gubba S.R., Tushar W., Yuen C., Hassan N.U., Poor H., Wood K.L., Wen C.K. (2017). Data Driven Electricity Management for Residential Air Conditioning Systems: An Experimental Approach. IEEE Trans. Emerg. Top. Comput..

[16] Miller C., Meggers F.M. (2017). The Building Data Genome Project: An open, public data set from non-residential buildings electrical meters. Energy Procedia.

[17] Cornell University Facilities Services Energy and Sustainability portal EMCS: Real Time Building Utility Use Data. http://portal.emcs.cornell.edu/.

[18] Baccarelli E., Naranjo P., Scarpiniti M., Shojafar M., Abawajy J. (2017). Fog of Everything: Energy-Efficient Networked Computing Architectures, Research Challenges, and a Case Study. IEEE Access.

[19] Amaxilatis D., Akrivopoulos O., Chatzigiannakis I., Tselios C. Enabling Stream Processing for People-Centric IoT Based on the Fog Computing Paradigm. Proceedings of the 22nd IEEE International Conference on Emerging Technologies and Factory Automation, ETFA 2017.

[20] Fotopoulou E., Zafeiropoulos A., Terroso-Sáenz F., Şimşek U., González-Vidal A., Tsiolis G., Gouvas P., Liapis P., Fensel A., Skarmeta A. (2017). Providing Personalized Energy Management and Awareness Services for Energy Efficiency in Smart Buildings. Sensors.

[21] Pocero L., Amaxilatis D., Mylonas G., Chatzigiannakis I. (2017). Open source IoT meter devices for smart and energy-efficient school buildings. HardwareX.

[22] Cuffaro G., Paganelli F., Mylonas G. A Resource-based Rule Engine for energy savings recommendations in Educational Buildings. Proceedings of the Global Internet of Things Summit 2017.

[23] Batra N., Singh A., Whitehouse K. (2015). If You Measure It, Can You Improve It? Exploring The Value of Energy Disaggregation. Proceedings of the 2nd ACM International Conference on Embedded Systems for Energy-Efficient Built Environments.

[24] Ardakanian O., Bhattacharya A., Culler D. (2016). Non-Intrusive Techniques for Establishing Occupancy Related Energy Savings in Commercial Buildings. Proceedings of the 3rd ACM International Conference on Systems for Energy-Efficient Built Environments.

[25] Li W., Yuen C., Hassan N.U., Tushar W., Wen C., Wood K.L., Hu K., Liu X. (2015). Demand Response Management for Residential Smart Grid: From Theory to Practice. IEEE Access.

[26] Theodoridis E., Mylonas G., Chatzigiannakis I. Developing an IoT Smart City framework. Proceedings of the 2013 Fourth International Conference on Information, Intelligence, Systems and Applications (IISA).

[27] Chatzigiannakis I., Mylonas G., Vitaletti A. (2011). Urban pervasive applications: Challenges, scenarios and case studies. Comput. Sci. Rev..

[28] Chatzigiannakis I., Mylonas G., Nikoletseas S. 50 ways to build your application: A survey of middleware and systems for wireless sensor networks. Proceedings of the IEEE Conference on Emerging Technologies and Factory Automation (ETFA).

[29] Georgitzikis V., Akribopoulos O., Chatzigiannakis I. (2012). Controlling physical objects via the internet using the arduino platform over 802.15.4 networks. IEEE Latin Am. Trans..

[30] Chatzigiannakis I., Mylonas G., Nikoletseas S. JWebDust: A Java-based generic application environment for wireless sensor networks. Proceedings of the First IEEE international conference on Distributed Computing in Sensor Systems.

[31] Tushar W., Yuen C., Li K., Wood K.L., Zhang W., Liu X. (2016). Design of Cloud-Connected IoT System for Smart Buildings on Energy Management (Invited Paper). EAI Endorsed Trans. Ind. Netw. Intellig. Syst..

[32] Withanage C., Ashok R., Yuen C., Otto K. A comparison of the popular home automation technologies. Proceedings of the 2014 IEEE Innovative Smart Grid Technologies—Asia (ISGT Asia).

[33] Fasolo E., Rossi M., Widmer J., Zorzi M. (2007). In-Network Aggregation Techniques for Wireless Sensor Networks: A Survey. Wireless Commun..

[34] Boavida F., Kliem A., Renner T., Riekki J., Jouvray C., Jacovi M., Ivanov S., Guadagni F., Gil P., Triviño A., Novais P., Camacho D., Analide C., El Fallah Seghrouchni A., Badica C. (2016). People-Centric Internet of Things—Challenges, Approach, and Enabling Technologies. Intelligent Distributed Computing IX: Proceedings of the 9th International Symposium on Intelligent Distributed Computing – IDC’2015, Guimarães, Portugal, October 2015.

[35] Y.4000/Y.2060, I.T. (2012). Overview of the Internet of Things.

[36] Ardakanian O., Bhattacharya A., Culler D. Non-intrusive techniques for establishing occupancy related energy savings in commercial buildings. Proceedings of the 3rd ACM International Conference on Systems for Energy-Efficient Built Environments.

[37] Card S.K., Robertson G.G., Mackinlay J.D. (1991). The Information Visualizer, an Information Workspace. Proceedings of the SIGCHI Conference on Human Factors in Computing Systems.

[38] Zacharioudakis E., Leligou H.C., Papadopoulou A. Energy efficiency tools for residential users. Proceedings of the 21st International Conference on Circuits, Systems, Communications and Computers.

